# Ferroptosis vulnerability in MYCN‐driven neuroblastomas

**DOI:** 10.1002/ctm2.963

**Published:** 2022-07-31

**Authors:** Shayani Dasgupta, Boyi Gan

**Affiliations:** ^1^ Department of Experimental Radiation Oncology The University of Texas MD Anderson Cancer Center Houston Texas USA; ^2^ The University of Texas MD Anderson UTHealth Graduate School of Biomedical Sciences Houston Texas USA

1

Resistance to cell death is an important hallmark of cancer.^1^ Over the past two decades, researchers have been developing therapeutic strategies to target cell death signalling pathways for cancer treatment. In recent years, ferroptosis, an iron‐dependent non‐apoptotic form of regulated cell death, has gained enormous attention in cancer research fields, because ferroptosis has been demonstrated to play a pivotal role in tumour suppression and because targeting ferroptosis represents a promising strategy in cancer therapy.^2^ Ferroptosis is driven by excessive accumulation of lipid peroxides on cellular membranes, which results from an imbalance in cellular redox state due to increased levels of reactive oxygen species (ROS) and/or inactivation of cellular antioxidant systems.^3^ Protection against ferroptosis largely depends on the antioxidant system mediated by the glutathione (GSH)‐glutathione peroxidase 4 (GPX4) axis, wherein cystine uptake via the cystine/glutamate antiporter (xCT, also called SLC7A11) promotes the synthesis of intracellular GSH,^4^, ^5^ which is further used as a cofactor by GPX4 to catalyse the reduction of lipid peroxides to non‐toxic lipid alcohols.^6^

Abnormal metabolism is a salient feature of cancer cells.^1^ To maintain nutrient demands for uncontrolled cell growth and proliferation, cancer cells reprogram a variety of metabolic pathways through regulating multiple oncogenic and tumour suppression factors. The MYC family of oncoproteins, including MYC, MYCL and MYCN, are ‘super‐transcription factors’ that mediate transcription of many metabolic genes and govern cellular metabolism in cancer cells. Majority of human cancers exhibit increased expression of MYC and its family members, leading to global metabolic reprogramming, which in turn provides energy sources and redox potential to support uncontrolled proliferation of cancer cells.^7^

Considering that the MYC family plays a key role in regulating cancer metabolism and cell death/survival, exploiting the link between MYC and metabolic cell death pathways, such as ferroptosis, might provide new approaches in treating cancers with aberrant MYC expression. In a recent study published in *Nature Cancer*, Alborzinia et al. identified a previously unappreciated role of MYCN in regulating ferroptosis sensitization.^8^ The authors chose childhood neuroblastoma as a model to study MYCN‐driven cancers.^9^ In their initial exploration, the authors^8^ observed a link between MYCN expression and intracellular cysteine/GSH levels. Intracellular cysteine and GSH pools are known to suppress ferroptosis, and depletion of GSH or cystine (the oxidized dimeric form of cysteine) effectively sensitizes cancer cells to ferroptosis.^5^ The data obtained by Alborzinia et al. confirmed the dependency of cystine starvation‐induced cell death on MYCN expression, as cell death caused by cystine deprivation could be rescued by suppressing MYCN expression or activity. Abrogation of cell death induced by cystine depletion in MYCN‐expressing neuroblastoma cells upon treatment with ferroptosis inhibitors or iron chelators further support their hypothesis that MYCN regulates ferroptosis.^8^

The authors then performed unbiased small interfering RNA (siRNA) screenings in matched MYCN‐high versus ‐low cells to identify high MYCN‐associated cellular vulnerabilities to ferroptosis. Their data revealed that enzymes involved in GSH metabolism and biosynthesis, such as *GSR*, *GPX4*, *GPX6*, *GSTM1*, *GSTM5* and *GSTK1*, are relatively more essential for cell viability in MYCN‐high cells than in MYCN‐low counterparts. Consistently, *GPX4* knockdown or GPX4 inactivation by its inhibitor RSL3 induced much more ferroptosis in MYCN‐high cells than in MYCN‐low cells.^8^ Furthermore, inducible ablation of *GPX4* in vivo suppressed the growth of MYCN‐amplified neuroblastomas. Considering that GPX4 is an essential gene in many cancer cell lines,^6^ it will be important to assess whether GPX4 ablation also affects the growth of corresponding MYCN‐low neuroblastomas (and therefore whether GPX4 inactivation would *selectively* impair MYCN‐high tumour growth).

While xCT‐mediated cystine uptake provides the major source for intracellular cysteine pools, cancer cells can also obtain cysteine from alternative pathways such as de novo cysteine synthesis through the transsulfuration pathway (intracellular conversion of methionine to cysteine).^10^ Alborzinia et al. observed an upregulation of transsulfuration with increased MYCN expression.^8^ In the transsulfuration pathway, homocysteine produced from the methionine cycle combines with serine to form cystathionine by cystathionine beta‐synthase (CBS); cystathionine is subsequently converted to cysteine by cystathionine gamma lyase (CTH).^10^ The authors further showed that supplementation with homocysteine/cystathionine prevented ferroptosis in cysteine‐depleted MYCN‐high adrenergic neuroblastoma cells (which maintain active transsulfuration pathway), but not in cysteine‐depleted MYCN‐high mesenchymal neuroblastoma cells (with inactive transsulfuration pathway), signifying that activated transsulfuration pathway is important for maintaining intracellular cysteine levels for GSH‐mediated protection against ferroptosis. Likewise, CTH inhibition exerted more ferroptosis‐sensitizing effects on MYCN‐high adrenergic neuroblastoma cells than on MYCN‐high mesenchymal neuroblastoma cells. Further studies by the authors suggested that, in MYCN‐high adrenergic neuroblastoma cells, upregulated transsulfuration pathway maintains the source of intracellular cysteine for protein synthesis at the expense of GSH synthesis, thus triggering a ferroptosis vulnerability.

These cell line studies suggest a unique MYCN‐dependent metabolic rewiring that might create a new vulnerability for therapeutically targeting in MYCN‐high adrenergic neuroblastomas. The authors tested the therapeutic implication of their findings in vivo using an orthotopic MYCN‐driven neuroblastoma model. Intriguingly, 60% reduction in tumour growth was observed upon pharmacological inhibition of both cystine uptake and cysteine synthesis via transsulfuration. Furthermore, a remarkable remission in tumour growth was observed upon combining this approach in *GPX4* knockout tumours. Cumulatively, their data support a triple combination therapy by inhibiting GPX4, cystine uptake and cysteine synthesis to eliminate MYCN‐amplified neuroblastomas.

Collectively, these findings identify cysteine addiction as a new liability in MYCN‐amplified neuroblastomas and propose to use ferroptosis‐inducing therapeutic strategies to treat MYCN‐driven neuroblastomas (Figure 1). However, several important questions remain to be addressed in order to translate these exciting findings into clinic. First, the current study (like several previous studies) used genetic ablation of GPX4 as the approach to inhibit GPX4 in tumours, because currently there is no good GPX4 inhibitor available that is suitable for in vivo treatment. Therefore, once such GPX4 inhibitors are available in the near future, additional preclinical studies with GPX4 inhibitor treatment are required to further validate their findings. In addition, combining GPX4 inhibition with blockade of both xCT and transsulfuration is likely to be also toxic in normal cells and tissues. Careful analyses of normal tissues with these treatments in preclinical studies are required in future investigations. Finally, a comparison between MYCN‐high and ‐low tumour models with these treatments will help illuminate whether ferroptosis indeed represents a selective “Achilles' heel” for MYCN‐high tumours (or the triple combination can universally suppress tumour formation regardless of MYCN status). Undoubtedly, this study will inspire further studies to explore ferroptosis as a vulnerability in cancer therapy.

**FIGURE 1:**
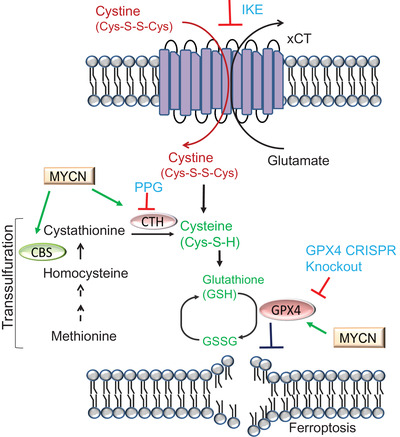
Schematic shows combined inhibition of cystine uptake (by IKE), cysteine de novo synthesis (by propargylglycine [PPG] to block CTH, a key enzyme involved in cysteine synthesis) and GPX4 (by *GPX4* CRISPR knockout) depletes intracellular cysteine and GSH availability, and effectively triggers ferroptosis in MYCN‐high neuroblastomas.

## CONFLICT OF INTEREST

Boyi Gan is an inventor of patent applications involving targeting ferroptosis in cancer therapy. Shayani Dasgupta has no conflicts of interest to declare.
